# A Population Pharmacokinetic Model-Guided Evaluation of Ceftolozane-Tazobactam Dosing in Critically Ill Patients Undergoing Continuous Venovenous Hemodiafiltration

**DOI:** 10.1128/AAC.01655-19

**Published:** 2019-12-20

**Authors:** Fekade B. Sime, Melissa Lassig-Smith, Therese Starr, Janine Stuart, Saurabh Pandey, Suzanne L. Parker, Steven C. Wallis, Jeffrey Lipman, Jason A. Roberts

**Affiliations:** a University of Queensland Centre for Clinical Research, Faculty of Medicine, The University of Queensland, Brisbane, Australia; b School of Pharmacy, Centre for Translational Anti-infective Pharmacodynamics, The University of Queensland, Brisbane, Australia; c Department of Intensive Care Medicine, Royal Brisbane and Women’s Hospital, Brisbane, Australia; d Division of Anaesthesiology Critical Care Emergency and Pain Medicine, Nîmes University Hospital, University of Montpellier, Nîmes, France; e Pharmacy Department, Royal Brisbane and Women’s Hospital, Brisbane, Australia

**Keywords:** ceftolozane-tazobactam, pharmacokinetics, renal replacement therapy, hemodiafiltration, CRRT

## Abstract

The aim of this work was to describe optimized dosing regimens of ceftolozane-tazobactam for critically ill patients receiving continuous venovenous hemodiafiltration (CVVHDF). We conducted a prospective observational pharmacokinetic study in adult critically ill patients with clinical indications for ceftolozane-tazobactam and CVVHDF. Unbound drug concentrations were measured from serial prefilter blood, postfilter blood, and ultrafiltrate samples by a chromatographic assay. Population pharmacokinetic modeling and dosing simulations were performed using Pmetrics. A four-compartment pharmacokinetic model adequately described the data from six patients. The mean (± standard deviation [SD]) extraction ratios for ceftolozane and tazobactam were 0.76 ± 0.08 and 0.73 ± 0.1, respectively. The mean ± SD sieving coefficients were 0.94 ± 0.24 and 1.08 ± 0.30, respectively. Model-estimated CVVHDF clearance rates were 2.7 ± 0.8 and 3.0 ± 0.6 liters/h, respectively. Residual non-CVVHDF clearance rates were 0.6 ± 0.5 and 3.3 ± 0.9 liters/h, respectively. In the initial 24 h, doses as low as 0.75 g every 8 h enabled cumulative fractional response of ≥85% for empirical coverage against Pseudomonas aeruginosa, considering a 40% *fT*_>MIC_ (percentage of time the free drug concentration was above the MIC) target. For 100% *fT*_>MIC_, doses of at least 1.5 g every 8 h were required. The median (interquartile range) steady-state trough ceftolozane concentrations for simulated regimens of 1.5 g and 3.0 g every 8 h were 28 (21 to 42) and 56 (42 to 84) mg/liter, respectively. The corresponding tazobactam concentrations were 6.1 (5.5 to 6.7) and 12.1 (11.0 to 13.4) mg/liter, respectively. We suggest a front-loaded regimen with a single 3.0-g loading dose followed by 0.75 g every 8 h for critically ill patients undergoing CVVHDF with study blood and dialysate flow rates.

## Abstract (precis)

The aim of this work was to describe optimized dosing regimens of ceftolozane-tazobactam for critically ill patients receiving continuous venovenous hemodiafiltration (CVVHDF). We conducted a prospective observational pharmacokinetic study in adult critically ill patients with clinical indications for ceftolozane-tazobactam and CVVHDF. Unbound drug concentrations were measured from serial prefilter blood, postfilter blood, and ultrafiltrate samples by a chromatographic assay.

## TEXT

Acute kidney injury (AKI) is a common complication of sepsis necessitating the use of renal replacement therapy (RRT) (1). RRT is delivered either intermittently or continuously. In critically ill patients whose condition is unstable, continuous RRT (CRRT) in the form of continuous venovenous hemofiltration (CVVHF) or continuous venovenous hemodiafiltration (CVVHDF) is commonly used for better fluid control and hemodynamic stability (2, 3). The CVVHDF modality of CRRT is commonly used in some parts of the world (e.g., 54% in Australian and New Zealand intensive care units [ICUs]) (4).

Antibiotic dosing in critically ill patients undergoing CRRT is considered challenging. The extent of total drug clearance (CL) during CRRT is variable not only due to the different modalities and operational settings of CRRT used across different institutions but also due to the variable residual renal and nonrenal clearance pathways (5). The traditional dosing considerations in patients undergoing CRRT mainly focus on the notion of renal impairment and generally result in low doses without appropriately accounting for the substantial extracorporeal clearance and thus for the risk of underdosing (6–8). The risk of underdosing is particularly high in the initial phase of treatment compared to later in the course of therapy when the drug may accumulate to provide high exposure (9). However, inadequate antibiotic exposure during the initial critical phase of therapy is highly likely to result in treatment failure.

In a recent study (10), the use of CRRT was identified as an independent risk factor for treatment failure of ceftolozane-tazobactam in the treatment of Pseudomonas aeruginosa infections. Although the authors did not investigate why treatment failure was high during CRRT, they alluded to the fact that there is no clearly defined dosing recommendation for ceftolozane-tazobactam in the different forms of CRRT. In their study, all patients received intermittent infusion of 1.5 g every 8 h (g8h), and the authors suggested increasing the dose to minimize risk of treatment failure. However, with the exception of a few case reports (11–13), there are limited data to address the issue of whether underdosing was the reason for the increased treatment failure during CRRT or if higher doses achieve appropriate exposure without risking unnecessary accumulation of the drug.

The aim of this work was, therefore, to describe optimized dosing regimens of ceftolozane-tazobactam in critically ill patients receiving CVVHDF based on a population pharmacokinetic (PK) model developed from simultaneous analysis of unbound concentrations in prefilter patient plasma, postfilter plasma, and RRT effluent during CVVHDF.

## RESULTS

Demographic and clinical characteristics of each study participant are given in Table 1. All patients received the CVVHDF mode of RRT. The RRT settings for each participant are summarized in Table 2. The mean (± standard deviation [SD]) extraction ratios (ERs) for ceftolozane and tazobactam were 0.76 ± 0.08 and 0.73 ± 0.1, respectively. The mean ± SD sieving coefficients (SCs) were 0.94 ± 0.24 and 1.08 ± 0.30, respectively. CVVHDF clearances estimated by the noncompartmental method were 2.92 ± 0.6 liters/h and 2.85 ± 0.6 liters/h ceftolozane and tazobactam, respectively.

**TABLE 1:** Demographic and clinical characteristics of study participants^*a*^

Patient no.	Sex	Age (yrs)	Wt (kg)	Serum creatinine (μmol/liter)	Albumin (g/liter)	ALT (IU/ml)	AST (IU/ml)	ALP (IU/ml)	Bilirubin (μmol/liter)	APACHE II score	SOFA score	Site(s) of infection	Organism(s)
1	Male	23	65	137	27	33	35	134	13	40	7	Blood + lung	Carbapenem-resistant Pseudomonas aeruginosa, Serratia marcescens, Klebsiella pneumoniae
2	Male	66	65	156	32	1440	762	134	55	29	13	Blood	P. aeruginosa
3	Female	65	80	77	25	44	78	126	10	37	14	Unknown	Stenotrophomonas maltophilia,^*b*^ Candida albicans
4	Male	65	103	139	24	39	68	195	36	22	6	Blood + lung	S. marcescens, Staphylococcus epidermidis
5	Male	58	65	75	26	35	19	116	32	35	10	Blood	Staphylococcus haemolyticus, Enterococcus faecium
6	Male	58	100	272	29	131	569	125	253	24	16	Lung	Stenotrophomonas maltophilia

Median		61.5	72.5	138	26.5	41.5	73	130	34	32	11.5		
Q1		58	65	92	25.25	36	43.25	125.25	17.75	25.25	7.75		
Q3		65	95	151.75	28.5	109.25	446.25	134	50.25	36.5	13.75		

a ALT, alanine aminotransferase; AST, aspartate aminotransferase; ALP, alkaline phosphatase; APACHE II, acute physiology and chronic health evaluation II; SOFA, sequential organ failure assessment; Q1, first quartile; Q3, third quartile.

b Suspected.

**TABLE 2:** Renal replacement therapy settings for the study participants^*a*^

Patient no.	Filter type	Filtration time (h)	Blood flow rate (ml/min)	Dialysate flow rate (ml/h)	Prefilter dilution (ml/h)	Postfilter dilution (ml/h)	Target fluid removal (ml/h)	Hematocrit
1	ST100	14	150	1,000	1,800	200	70	0.22
2	ST150	15	100	1,000	1,167	500	220	0.3
3	ST100	38	150	1,500	200		100	0.3
4	ST150	31	100	1,500	1,000	1,500	30	0.24
5	ST100	46	200	1,500	500		100	0.25
6	ST150	9	200	1,000	1,800		500	0.23

a For ST100 and AN69 (acrylonitrile and sodium-methallylsulfonate copolymer), a hemofilter with a surface area of 1 m^2^ was used; for ST150 and AN69, a hemofilter with a surface area of 1.5 m^2^ was used.

**FIG 1:**
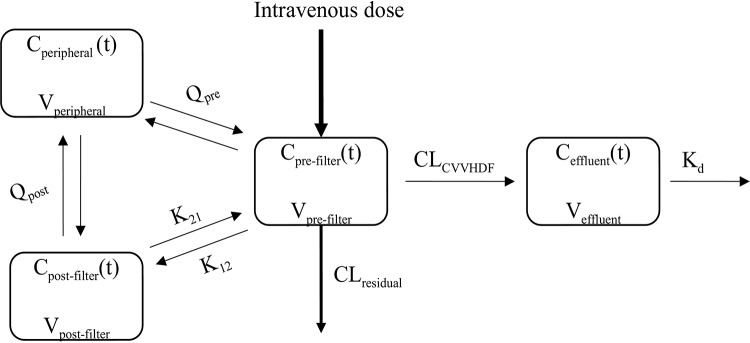
Schematics of the structural pharmacokinetic model. C_pre-filter_ (t), concentration in the prefilter compartment at time *t*; C_post-filter_ (t), concentration in the postfilter compartment at time *t*; C_peripheral_ (t), concentration in the peripheral compartment at time *t*; C_effluent_ (t), concentration in the effluent compartment at time *t*; CL_CVVHDF_, clearance by continuous venovenous hemodiafiltration; CL_residual_, residual non-CVVHDF clearance; V_post_, volume of the postfilter compartment; V_pre_, volume of the prefilter compartment; K_12_, rate constant for transfer from the prefilter compartment to the postfilter compartment; K_21_, rate constant for transfer from the postfilter compartment to the prefilter compartment; K_d_, rate constant for transfer out of the effluent compartment (“drainage”); V_effluent_, volume of the effluent compartment; Q_pre_, intercompartmental clearance between the prefilter compartment and the peripheral compartment; Q_post_, intercompartmental clearance between the postfilter compartment and the peripheral compartment; V_peripheral_, volume of the peripheral compartment.

**FIG 2:**
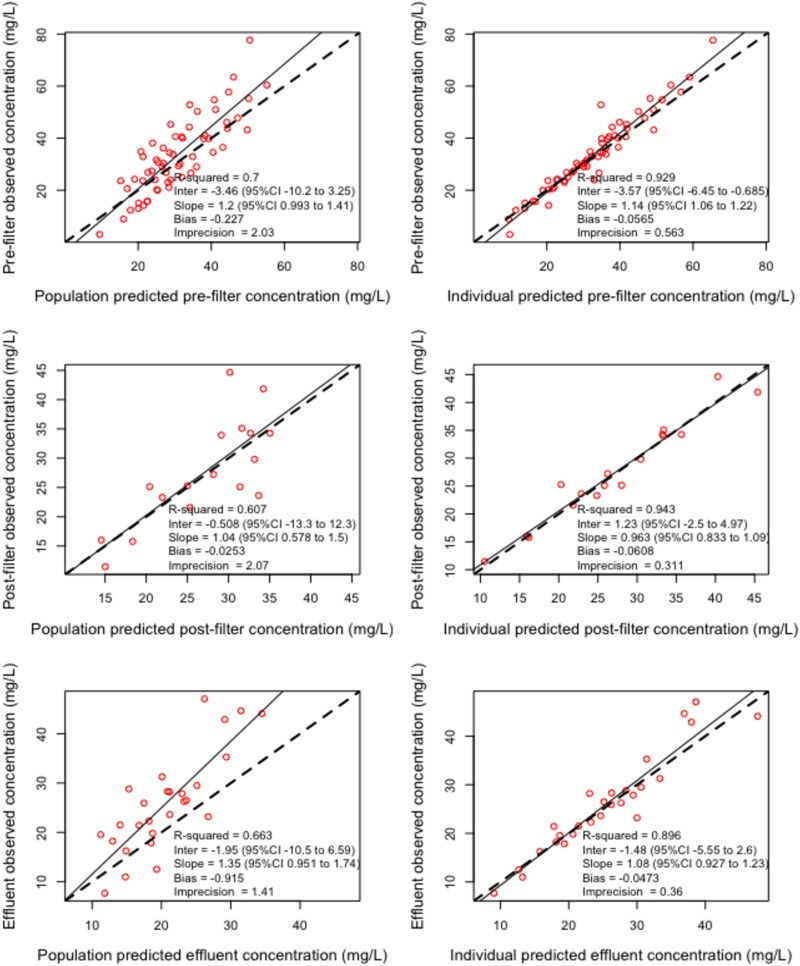
Observed-versus-predicted concentration diagnostic plots for ceftolozane. 95%CI, 95% confidence interval; Inter, intercept.

**FIG 3:**
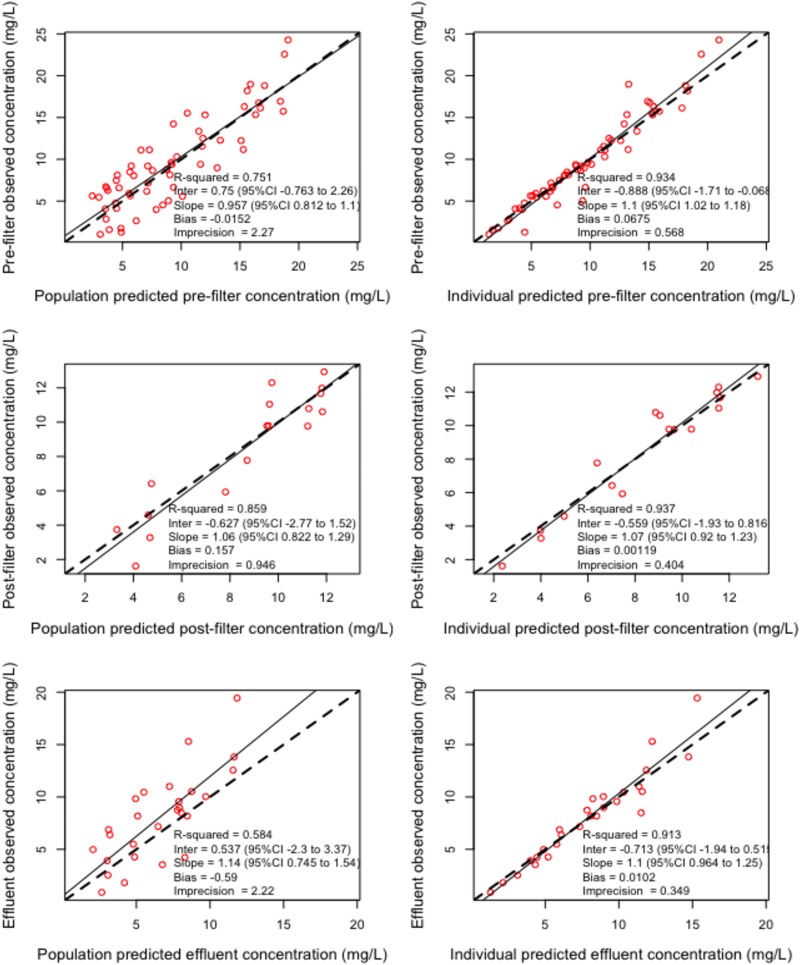
Observed-versus-predicted concentration diagnostic plots for tazobactam. 95%CI, 95% confidence interval; Inter, intercept.

A four-compartment model schematically described in Fig. 1, with CVVHDF and non-CVVHD residual clearance from a prefilter central compartment, adequately described the data. This final structural model was used for dosing simulations as none of the available covariates improved model fit. The observed versus predicted plots for ceftolozane and tazobactam are depicted in Fig. 2 and 3, respectively. The visual predictive check plots for prefilter patient plasma concentrations of both ceftolozane and tazobactam are shown in Fig. S1 in the supplemental material. Parameter estimates for the final models are given in Table 3. Model-predicted CVVHDF filter clearance rates were similar to values determined from measured cumulative amounts of the drugs in effluent bags.

**TABLE 3:** Parameter estimates for the final ceftolozane and tazobactam models^*a*^

Parameter (unit)	Value					
	Ceftolozane		Shrink rate (%)	Tazobactam		Shrink rate (%)
	Mean	SD		Mean	SD	
CL_CVVHDF_ (liters/h)	2.659	0.783	0.00	2.973	0.603	0.06
CL_residual_ (liters/h)	0.596	0.504	0.00	3.254	0.867	0.011
*V*_post_ (liters)	17.578	10.871	0.00	19.685	14.382	0.010
*V*_pre_ (liters)	25.184	7.499	0.00	28.206	6.603	0.126
*K*_12_ (h^−1^)	0.43	0.718	0.00	0.561	0.638	0.092
*K*_21_ (h^−1^)	0.676	0.908	0.00	1.01	1.01	0.013
*K_d_* (h^−1^)	1.596	0.495	0.00	1.584	0.483	0.013
*V*_effluent_ (liters)	2.178	0.801	0.00	2.609	1.561	0.006
*Q*_pre_ (liters/h)	0.834	1.863	0.00	2.547	2.455	0.009
*Q*_post_ (liters/h)	2.42	1.451	0.00	3.612	1.204	0.005
*V*_peripheral_ (liters)	73.379	39.042	0.00	77.196	32.267	0.011

a SD, standard deviation; CL_CVVHDF_, clearance by continuous venovenous hemodiafiltration; CL_residual_, residual non-CVVHDF clearance; *V*_post_, volume of the postfilter compartment; *V*_pre_, volume of the prefilter compartment; *K*_12_, rate constant for transfer from the prefilter compartment to the postfilter compartment; *K*_21_, rate constant for transfer from the postfilter compartment to the prefilter compartment; *K_d_*, rate constant for transfer out of the effluent compartment (“drainage”); *V*_effluent_, volume of the effluent compartment; *Q*_pre_, intercompartmental clearance between the prefilter and the peripheral compartments; *Q*_post_, intercompartmental clearance between the postfilter and peripheral compartments; *V*_peripheral_, volume of the peripheral compartment.

For the pharmacokinetic (PK)/pharmacodynamic (PD) target of 40% *fT*_>MIC_ (percentage of time the free drug concentration was above the MIC), all simulated dosing regimens achieved a probability of target attainment (PTA) of ≥0.9 for MIC values in the susceptible range (≤4 mg/liter) during the first 24 h of treatment. In addition, at PK steady state, all simulated dosing regimens achieved a PTA of ≥0.9 for all targets when the MIC values were ≤4 mg/liter (susceptible). However, during the first 24 h after commencement of dosing, for higher PK/PD targets of 60% and 100% *fT*_>MIC_, intermittent doses of ≥0.75 g every 8 h (q8h) and 1.5 g q8h, respectively, and continuous infusion doses of ≥0.375 g loading dose (LD) plus 1.125 g continuous infusion and 1.5 g LD plus 4.5 g continuous infusion, respectively, were required to achieve PTA of ≥0.9 for MIC values in the susceptible range (≤4 mg/liter). Extended infusion without a loading dose resulted in significantly lower PTA during the first 24 h than was seen with the corresponding intermittent infusion across all doses investigated.

Table 4 shows the cumulative fractional response (CFR) for ceftolozane against P. aeruginosa EUCAST MIC distributions for exposure during the first 24 h of treatment. For the PK/PD target of 40% *fT*_>MIC_, doses as low as 0.75 g q8h achieved optimal (≥85%) CFR for empirical therapy. However, for the higher target of 100% *fT*_>MIC_, 1.5 g q8h, 3.0 g q8h, or 3.0 g LD plus 9.0 g continuous infusion or 1.5 g LD plus 4.5 g continuous infusion was required to achieve optimal CFR for empirical therapy. For directed therapy, on the other hand, all simulated doses achieved optimal CFR for up to 60% *fT*_>MIC_, and doses as low as 0.75 g q8h achieved optimal CFR for 100% *fT*_>MIC_. At steady state (data not shown), all simulated doses achieved ≥85% CFR for empirical therapy and 100% CFR for directed therapy.

**TABLE 4:** Cumulative fractional response against Pseudomonas aeruginosa EUCAST MIC distribution for exposure during the first 24 h of treatment^*a*^

Dosing regimen	CFR rate by PK/PD target for empirical therapy			CFR rate by PK/PD target for directed therapy		
	40 %*fT*_>MIC_	60 %*fT*_>MIC_	100 %*fT*_>MIC_	40 %*fT*_>MIC_	60 %*fT*_>MIC_	100 %*fT*_>MIC_
0.375 g q8h	0.82	0.79	0.65	0.99	0.96	0.80
0.375 g 4 h EI q8h	0.82	0.79	0.24	0.99	0.96	0.29
0.375 g LD + 1.125 g continuous infusion	0.83	0.83	0.68	1.00	1.00	0.83
0.75 g q8h	0.86	0.84	0.77	1.00	1.00	0.94
0.75 g 4 h EI q8h	0.86	0.86	0.51	1.00	1.00	0.62
0.75 g LD + 2.25 g continuous infusion	0.86	0.86	0.79	1.00	1.00	0.96
1.5 g q8h	0.87	0.87	0.85	1.00	1.00	1.00
1.5 g 4 h EI q8h	0.87	0.87	0.69	1.00	1.00	0.84
1.5 g LD + 4.5 g continuous infusion	0.87	0.87	0.86	1.00	1.00	1.00
1.5 g LD for 24 h + 0.75 g q8h	0.87	0.87	0.85	1.00	1.00	1.00
3.0 g q8h	0.90	0.88	0.86	1.00	1.00	1.00
3.0 g 4 h EI q8h	0.90	0.88	0.79	1.00	1.00	0.96
3.0 g LD + 9.0 g continuous infusion	0.92	0.91	0.87	1.00	1.00	1.00
3.0 g LD + 0.75 g q8h	0.87	0.87	0.86	1.00	1.00	1.00

a PK, pharmacokinetic; PD, pharmacodynamic; CFR, cumulative fractional response; EI, extended infusion; q8h, every 8 h intermittent infusion (1 h); % *fT*_>MIC_, percentage of time free drug concentration was above the MIC; LD, loading dose over 1 h; continuous infusion, continuous infusion over 24 h.

Table S1 in the supplemental material summarizes the probability of achieving selected tazobactam exposures of 20% *fT*_>1mg/liter_, 50% *fT*_>2mg/liter_, and 100% *fT*_>4mg/liter_ during the first 24 h of dosing and at steady state. All simulated doses achieved 20% *fT*_>1mg/liter_. However, at least 0.75 g q8h was required for 50% *fT*_>2mg/liter_ exposure. On the other hand, at least 1.5 g q8h and 3.0 g LD plus 9.0 g continuous infusion were required for 100% *fT*_>4mg/liter_ exposures at steady state and during the first 24 h, respectively.

The maximum concentrations of ceftolozane and tazobactam achieved at steady state from various simulated dosing regimens of ceftolozane-tazobactam in a virtual population of critically ill patients (*n* = 1,000) receiving CVVHDF are summarized in Table 5. For both ceftolozane and tazobactam, doubling the dose resulted in doubling the steady-state concentration.

**TABLE 5:** Maximum concentrations of ceftolozane and tazobactam achieved at steady state from various simulated dosing regimens of ceftolozane-tazobactam in virtual population of critically ill patients receiving continuous venovenous hemodiafiltration^*a*^

Dosing regimen	Median (IQR) steady-state ceftolozane concn (mg/liter) at:		Median (IQR) steady-state tazobactam concn (mg/liter) at:	
	End of infusion	Trough/*C*_ss_	End of infusion	Trough/*C*_ss_
0.375 g q8h	13 (12–19)	7 (5–10)	4.4 (4.1–5.1)	1.5 (1.3–1.8)
0.375 g 4 h EI q8h	11 (9–16)	8 (6–11)	3.1 (2.8–3.5)	1.8 (1.8–2.0)
0.375 g LD + 1.125 g continuous infusion		9 (7–13)		2.5 (2.2–2.8)
0.75 g q8h	27 (24–39)	14 (10–21)	8.8 (8.2–10.1)	3.0 (2.7–3.4)
0.75 g 4 h EI q8h	22 (18–31)	16 (12–23)	5.7 (6.1–7.0)	3.5 (3.2–3.9)
0.75 g LD + 2.25 g continuous infusion		18 (14–26)		5 (4.5–5.4)
1.5 g q8h	54 (47–78)	28 (21–42)	17.5 (16.4–20.2)	6.1 (5.5–6.7)
1.5 g 4 h EI q8h	44 (37–63)	31 (23–45)	12.3 (11.6–14)	7.0 (6.4–7.8)
1.5 g LD + 4.5 g continuous infusion		36 (30–53)		9.7 (9.1–10.8)
3.0 g q8h	107 (95–155)	56 (42–84)	35.0 (32.8–40.4)	12.1 (11.0–13.4)
3.0 g 4 h EI q8h	89 (74–126)	62 (47–90)	24.6 (23.3–28.0)	14.1 (12.9–15.5)
3.0 g LD + 9.0g continuous infusion		73 (59–106)		19.3 (18.2–21.6)

a IQR, interquartile range; *C*_ss_, steady-state concentration; q8h, every 8 h intermittent infusion (1 h); EI, extended infusion; LD, loading dose over 1 h; continuous infusion, continuous infusion over 24 h.

## DISCUSSION

This is the first report describing the unbound population pharmacokinetics of ceftolozane and tazobactam in critically ill patients undergoing CVVHDF. We observed sieving coefficients that are consistent with previous findings for continuous hemofiltration and dialysis (14). The observed extraction ratios for unbound ceftolozane (0.76 ± 0.08) and tazobactam (0.73 ± 0.1) were comparable to the values of 0.86 and 0.85, respectively, in a previous case report using continuous hemofiltration (11). The model-predicted CVVHDF clearance for ceftolozane (2.7 ± 0.8 liters/h) is also in agreement with the rate of 2.4 liters/h in a previous case report (12) for a patient with RRT settings comparable to those used for the patients in the current study (Table 2), i.e., blood flow rate of 200 ml/min, predilution rate of 1,000 ml/h, and postdilution rate of 750 ml/h. The total estimated ceftolozane clearance during CVVHDF (3.3 liters/h) was about half of the total ceftolozane clearance we recently described for critically ill patients without renal impairment (7.2 liters/h) (24).

The relatively low clearance (longer half-life) during CVVHDF results in a prolonged time-to pharmacokinetic steady state. Our dosing simulations revealed that the steady state was achieved only after 4 days for ceftolozane. This is important for dosing evaluation in that adequacy of exposure for PK/PD target attainment should be evaluated during the first 24 h of treatment together with assessment of extent of accumulation at steady state to avoid unnecessarily high concentrations that potentially risk toxicity. Based on the first 24 h of exposure and considering a 40% *fT*_>MIC_ target, 0.75 g q8h is adequate for empirical initiation of therapy (Table 4). For susceptible pathogens (MIC ≤ 4 mg/liter), this dose is also adequate to provide 100% *fT*_>MIC_ ceftolozane exposure. In addition, it achieves previously recommended tazobactam exposures of 20% *fT*_>1mg/liter_ and 50% *fT*_>2mg/liter_ (14, 15) (see Table S1 in the supplemental material). These results are concordant with an *in silico* simulation study based on *ex vivo* data that recommended 0.75 g q8h dosing as an optimal regimen for continuous hemofiltration and dialysis (14).

However, given that susceptibility data are usually not available at the initiation of therapy and that for the critically ill an initial empirical coverage at a higher target for ceftolozane (100% *fT*_>MIC_) is advantageous, our results (Table 4) suggest that a higher dose of 1.5 g q8h may be advantageous for initiation of therapy. The median (IQR) steady-state trough concentration with 1.5 g q8h dosing was 28 (range, 21 to 42) mg/liter. The concentrations seen are generally 5 to 10 times the MIC breakpoint for P. aeruginosa (4 mg/liter). Therefore, these concentrations are generally acceptable given that most experts consider trough concentrations above 10 times the MIC to represent a cutoff point for dose reduction of beta-lactam antibiotics, although not because of significant toxicity concerns but for avoidance of unnecessarily high exposures (16). In addition, keeping the concentration above 4 to 5 times the MIC has been shown to maximize the antibacterial effect of beta-lactams (17). Furthermore, given the poor reproducibility of MIC measurements, it is not uncommon that an isolate considered susceptible at the breakpoint MIC (4 mg/liter) is subsequently found to actually be resistant with retesting, with an MIC higher by up to two dilutions (up to 16 mg/liter) (18). Therefore, the steady-state concentrations achieved with 1.5 g q8h dosing are generally acceptable.

A 1.5 g LD followed by 4.5 g continuous infusion may also provide similar exposure, with an added advantage of avoiding the higher peak concentration seen with the intermittent regimen (Table 5) that may have no added benefit in maximizing efficacy. This continuous infusion regimen achieved a median steady-state concentration of 36 mg/liter, which is just below the 10× MIC cutoff point. In previous case reports, no ceftolozane-related adverse effects were observed at equivalent or higher concentrations (11, 13, 19). Thus, clinicians may choose to use continuous infusion if there is a particular clinical concern with increased peak concentrations during intermittent infusion. An alternative approach to ensure early exposure that maximizes efficacy and at the same time minimizes amount accumulating at steady state is to use a front-loaded intermittent regimen with 1.5 g q8h for the first 24 h followed by 0.75 g q8h; that approach resulted in a median steady-state trough concentration of 14 mg/liter in our dosing simulation studies. Another convenient approach is to use 3.0 g initial LD followed by 0.75 g q8h thereafter. These front-loaded regimens will ensure adequate initial exposure while minimizing unnecessary accumulation of the drug at steady state.

Although there is no clearly defined toxicity threshold for ceftolozane steady-state concentrations, a high dose of 3.0 g q8h appears to achieve unnecessarily high steady-state ceftolozane concentrations in all modes of delivery during CVVHDF (Table 5). Similarly high ceftolozane concentrations were observed in a case study of CVVHDF, with peak and trough total concentrations of 163.9 mg/liter (∼131 mg/liter unbound concentration) and 79.4 mg/liter (∼64 mg/liter unbound), respectively (12).

This study was not without limitations. First, the sample size of six patients was small. We acknowledge that this may have limited our ability to identify covariate relationships with model parameters given the limited spread of covariate values in the data set. Second, there was a lack of a well-defined target exposure, particularly for tazobactam. We used previously recommended targets of 20% *fT*_>1mg/liter_ and 50% *fT*_>2mg/liter_ (14, 15). However, these exposures are not concordant with the *in vitro* susceptibility testing protocol for beta-lactam/tazobactam combination antibiotics, where the tazobactam concentration is fixed at 4 mg/liter. This limits the ability to relate *in vitro* susceptibility (MIC values) to clinical exposure if the targets at which we aim (20% *fT*_>1mg/liter_ and 50% *fT*_>2mg/liter_) allow exposures of less than 4 mg/liter. Only 3.0 g q8h intermittent infusion or 3.0 g LD plus 9.0 g continuous infusion was able to achieve a high probability of greater than 4 mg/liter exposure during the first 24 h (Table S1). Finally, we acknowledge that the recommendations from this work relate only to similar CVVHDF settings (summarized in Table 2).

In conclusion, ceftolozane and tazobactam are efficiently cleared by CVVHDF albeit at a much lower rate than that seen with patients with normal renal function. A front-loaded intermittent regimen with a single 3.0 g LD followed by 0.75 g q8h or, alternatively, 1.5 g q8h for the first 24 h followed by 0.75 g q8h thereafter would be appropriate to achieve adequate initial exposure and minimize excessive drug accumulation at steady state.

## MATERIALS AND METHODS

### Study design and setting.

This was a prospective observational population pharmacokinetic study of ceftolozane-tazobactam in critically ill patients undergoing CRRT. The study was conducted at the University of Queensland Centre for Clinical Research. Patients were recruited from the Royal Brisbane and Women’s Hospital (RBWH) quaternary referral intensive care unit (ICU) (RBWH no. HREC/16/QRBW/211), and the University of Queensland human research ethics committees provided ethical clearance (no. 2016001368).

### Patients.

Adult patients (≥18 years) admitted to RBWH ICU who were prescribed CRRT were enrolled if diagnosed with systemic infection known or suspected to be caused by an organism susceptible to ceftolozane-tazobactam. Patients who were pregnant or had a documented or suspected allergy to penicillins and cephalosporin were excluded. Each study participant or his or her next of kin provided informed consent prior to enrollment.

### Ceftolozane-tazobactam dosing.

Per protocol, all patients received 1.5 g ceftolozane-tazobactam (2:1 ratio) administered every 8 h via intravenous infusion over 1 h. Any alternative initial dosing or dose adaptation deemed necessary by the attending clinicians was allowed.

### CRRT procedures.

The standard protocol for CRRT at the Royal Brisbane and Women Hospital was followed. The general CRRT modality at RBWH was CVVHDF using a Prismaflex hemodiafiltration machine (Gambro, Lund, Sweden) with an AN69 ST150 or ST100 polyacrylonitrile filter (Gambro, Lund, Sweden) (surface area of 1.50 m^2^ or 0.9 m^2^, respectively). The dialysis and replacement fluid was either Hemofiltration Solution 1 (HF1) (Gambro) or lactate-free Hemosol B0 (Gambro). Replacement fluid was administered both pre and postfilter or prefilter only. The blood flow rates were 100 to 200 ml/min. The dialysate flow rates were 1,000 to 1,500 ml/h. Replacement fluid rates were adjusted to each patient’s specific requirements.

### Sample collection.

Blood samples were collected pre- and postfiltration during a dosing interval in lithium-heparin blood collection tubes. Prefilter sampling times were just before the dose; during ceftolozane-tazobactam infusion at 15 min and 45 min; 15 min after the end of 1 h of infusion; at 2 h, 3 h, 4 h, 5 h, 6 h, and 7 h after the commencement of infusion; and at 8 h, just before the next dose. Postfilter samples were collected at 45 min, 2 h, and 6 h after the start of ceftolozane-tazobactam infusion. Ultrafiltrate samples from the effluent line were collected at 1 h, 2 h, 4 h, 6 h, and 8 h after the commencement of ceftolozane-tazobactam infusion. In addition, the ultrafiltrate volume in the effluent bag was measured at each of these time points with ultrafiltrate samples taken from the bag for drug concentration measurement.

### Ceftolozane and tazobactam assay.

Unbound concentrations of ceftolozane and tazobactam in plasma and renal replacement therapy effluent were measured by an ultra-high-performance liquid chromatography–tandem mass spectrometry (UHPLC-MS/MS) method on a Shimadzu Nexera2 UHPLC system coupled to a Shimadzu 8050 triple-quadrupole mass spectrometer (Kyoto, Japan). The unbound fraction of plasma was isolated by ultracentrifugation using Centrifree devices (Millipore, Tullagreen, Ireland). Sample (10 μl) was spiked with phosphate-buffered saline (pH 7.4) and internal standard (sulbactam plus l-cefazolin) and acetonitrile. The stationary phase was processed with a C_18_ Ultra IBD column (Restek, Bellefonte, USA) (100 by 2.1 mm, 3-μm pore size) operated at room temperature. Mobile phase A was 0.1% (vol/vol) formic acid–10 mM ammonium formate, and mobile phase B was 100% (vol/vol) acetonitrile–0.1% (vol/vol) formic acid. The mobile phase was delivered with gradient from 15% to 50% B at a flow rate of 0.3 ml/min for 5 min run time and produced a backpressure of approximately 2,800 lb/in^2^. Ceftolozane was monitored by positive-mode electrospray at multiple-reaction monitoring (MRM) values of 667.00 to 199.15. Labeled cefazolin was monitored in positive mode at 457.85 to 326.05. Tazobactam and sulbactam were monitored by negative-mode electrospray at MRM values of 299.20 to 138.00 and 232.20 to 140.00, respectively. The calibration range for ceftolozane was 1 to 100 mg/liter and for tazobactam was 0.5 to 100 mg/liter. For ceftolozane at total concentrations of 160, 20, and 3 mg/liter, the levels of precision of the unbound analysis were 6.3%, 6.2%, and 8.2% with unbound fractions of 90%, 99%, and 101%. For tazobactam at total concentrations of 80, 10, and 1.5 mg/liter, the levels of precision of unbound analysis were 6.2%, 7.5%, and 8.1% with unbound fractions of 89%, 91%, and 92%. The assay method was validated using the FDA criteria for bioanalysis (20).

### Pharmacokinetic analysis.

Initially noncompartmental analysis was performed to set the initial boundaries for relevant model parameters during subsequent population pharmacokinetic modeling. The extraction ratio (ER), sieving coefficient (SC), and extracorporeal clearance by the CVVHDF machine (CL_CVVHDF_) were determined based on observed concentrations using the following equations:

(1) $$\left(\begin{array}{c} \text{extraction ratio} \end{array}\right)=\frac{\text{concentration in postfilter blood sample}}{\text{concentration in prefilter blood sample}}$$

(2) (sieving coefficient)=effluent drug concentration[(prefilter plasmaconcentration)+(postfilter plasmaconcentration)]2

(3) $${\text{CL}}_{\text{CVVHDF}}=\frac{A_{\text{CVVHDF}}}{{\text{AUC}}_{0-8}}$$

where *A*_CVVHDF_ is the total amount of ceftolozane or tazobactam recovered in the ultrafiltrate and AUC_0–8_ is the area under the ultrafiltrate concentration-time curve determined by the linear trapezoidal rule.

Subsequently, a nonparametric population pharmacokinetic analysis was performed in R using the Pmetrics user interface to describe the unbound concentration-time profiles from prefilter plasma, postfilter plasma, and CVVHDF ultrafiltrate samples simultaneously. Three- and four-compartment models with first-order CVVHDF and residual non-CVVHDF clearance were tested. CVVDHF clearance was from the compartment representing prefilter samples. Residual clearance was tested on compartments representing both postfilter and prefilter samples. All between-compartment distributions were modeled as linear processes. Error models were based on standard deviations (SD) of observations (obs) available in Pmeterics as additive [error = (SD^2^ + λ^2^)^0.5^] and multiplicative (error = SD * γ) models, where λ and γ represent process noise. In addition, assay error was modeled with a first-degree polynomial function (error = C0 + C1*[obs]). Plausible clinical covariates were tested on residual non-CVVHDF clearance, intercompartmental clearances, and volumes of pre and postfilter compartments. Available covariates considered for analysis included sex, height, weight, body max index, body surface area, albumin concentration, serum creatinine, sequential organ failure assessment (SOFA) score, acute physiology and chronic health evaluation (APACHE) II score, dialysate flow rate, transmembrane pressure, filter type, and blood flow rate.

Models were evaluated by the combination of diagnostic goodness-of-fit plots and statistics. Diagnostic plots included scatterplots of observed-versus-predicted concentrations, visual predictive check plots, and normalized prediction distribution error (NPDE) versus time and output plots. Statistical evaluation of observed-versus-predicted concentrations was based regression coefficient *r*^2^, bias, and imprecision. In Pmetrics, bias is defined as the mean weighted error of predicted minus observed concentrations, Σ(predicted-observed/standard deviation)/N, and imprecision is defined as the bias-adjusted, mean weighted squared error of predicted minus observed concentration, i.e., Σ[(predicted-observed)^2^/(standard deviation)^2^]/*N* − Σ(predicted-observed)/standard deviations/*N*, where *N* is the number of observations/predictions. In addition, statistical model evaluation was performed based on objective function values that included the log-likelihood ratio (LLR), Akaike information criterion (AIC), and Bayesian information criterion (BIC). The LLR chi-square test within Pmetrics was used for statistical comparison of nested models (*P* < 0.05 was considered significant).

The final model was used to perform Monte Carlo dosing simulations (*n* = 1,000) and to assess the probability of target attainment (PTA) and extent of accumulation for selected dosing regimens. Simulated regimens included 0.75 g, 1.5 g, and 3.0 g ceftolozane-tazobactam (2:1 ratio) administered by 1 h intermittent infusion every 8 h (q8h), by 4 h extended infusion q8h, and by continuous infusion of the total daily dose following a single loading dose (LD) given over 1 h. Additional dosing regimens simulated included a front-loaded intermittent regimen of 1.5 g q8h for 24 h followed by 0.75 g q8h and a single 3.0 g LD followed by 0.75 g q8h. For ceftolozane, the primary target for PTA assessment was 40% *fT*_>MIC_, which is considered adequate for ∼1 log kill (21, 22). Secondary targets studied included 60% and 100% *fT*_>MIC_. For tazobactam, on the other hand, we performed assessments against previously suggested targets of 20% *fT*_>1mg/liter_ (20% of the time above minimum effective concentration of 1 mg/liter) (15) and 50% *fT*_>2mg/liter_ (14). In addition, given that the *in vitro* susceptibility of beta-lactam/tazobactam combination is tested fixing the tazobactam concentration at 4 mg/liter (23), we assessed attainment of 100% *fT*_>4mg/liter_. Prefilter patient plasma exposure was used for all PTA assessments.

CFR was estimated for ceftolozane, using the Pseudomonas aeruginosa EUCAST MIC distribution (accessed August 2019), for both empirical and directed therapy. A CFR value of ≥85% was considered acceptable. Equation 4 was used for CFR calculation as follows:

(4) $$\text{CFR}=\overset{n}{\underset{i=0.125}{\sum }}{\text{PTA}}_{i}\times F_{i}$$

where *i* is MIC category ranging from 0.125 to *n*, *n* is 64 mg/liter for empirical therapy and the EUCAST clinical breakpoint of 4 mg/liter for directed therapy, PTA*_i_* is the PTA at each MIC category, and *F_i_* is the fraction of the bacterial population for each MIC category.

## Supplementary Material

Supplemental file 1
