# Supplementary material for: A Population Pharmacokinetic Model-Guided Evaluation of Ceftolozane-Tazobactam Dosing in Critically Ill Patients Undergoing Continuous Venovenous Hemodiafiltration
Source: Antimicrob Agents Chemother. 2019 Dec 20;64(1):e01655-19. doi: 10.1128/AAC.01655-19 (PMC7187594; doi:10.1128/AAC.01655-19)

Supplemental Table 1. Probability of achieving selected tazobactam exposures during the first 24 hours of dosing and at steady state

Ceftolozane/tazobactam dosing regimen (2:1 ratio)	Probability of tazobactam exposure of 20% $fT_{>1mg/L}$		Probability of tazobactam exposure of 50% $fT_{>2mg/L}$		Probability of tazobactam exposure of 100% $fT_{>4mg/L}$	
	First 24 hours	At steady state	First 24 hours	At steady state	First 24 hours	At steady state
0.375 g q8h	1.0	1.0	0.1	0.8	0.0	0.0
0.375 g 4h EI q8h	1.0	1.0	0.1	1.0	0.0	0.0
0.375 g LD + 1.125 g CI	1.0	1.0	0.5	1.0	0.0	0.0
0.75 g q8h	1.0	1.0	1.0	1.0	0.0	0.0
0.75 g 4h EI q8h	1.0	1.0	1.0	1.0	0.0	0.1
0.75 g LD + 2.25 g CI	1.0	1.0	1.0	1.0	0.0	0.7
1.5 g q8h	1.0	1.0	1.0	1.0	0.0	1.0
1.5 g 4h EI q8h	1.0	1.0	1.0	1.0	0.0	1.0
1.5 g LD + 4.5g CI	1.0	1.0	1.0	1.0	0.5	1.0
1.5 g LD for 24h + 0.75 g q8h	1.0	1.0	1.0	1.0	0.0	0.1
3.0 g q8h	1.0	1.0	1.0	1.0	0.8	1.0
3.0 g 4h EI q8h	1.0	1.0	1.0	1.0	0.0	1.0
3.0 g LD+ 9.0 g CI	1.0	1.0	1.0	1.0	1.0	1.0
3.0 g LD + 0.75 g q8h	1.0	1.0	1.0	1.0	0.1	0.1

q8h, every eight hour intermittent infusion (1hour); EI, extended infusion; $\% fT_{>1mg/L \text{ or } 4mg/L}$, percentage of time free drug concentration is above the specified concentration; LD, loading dose over 1 hour; CI, continuous infusion over 24 hours.

Supplemental figure 1. Visual predictive check plots for ceftolozane and tazobactam pre-filter patient plasma concentrations

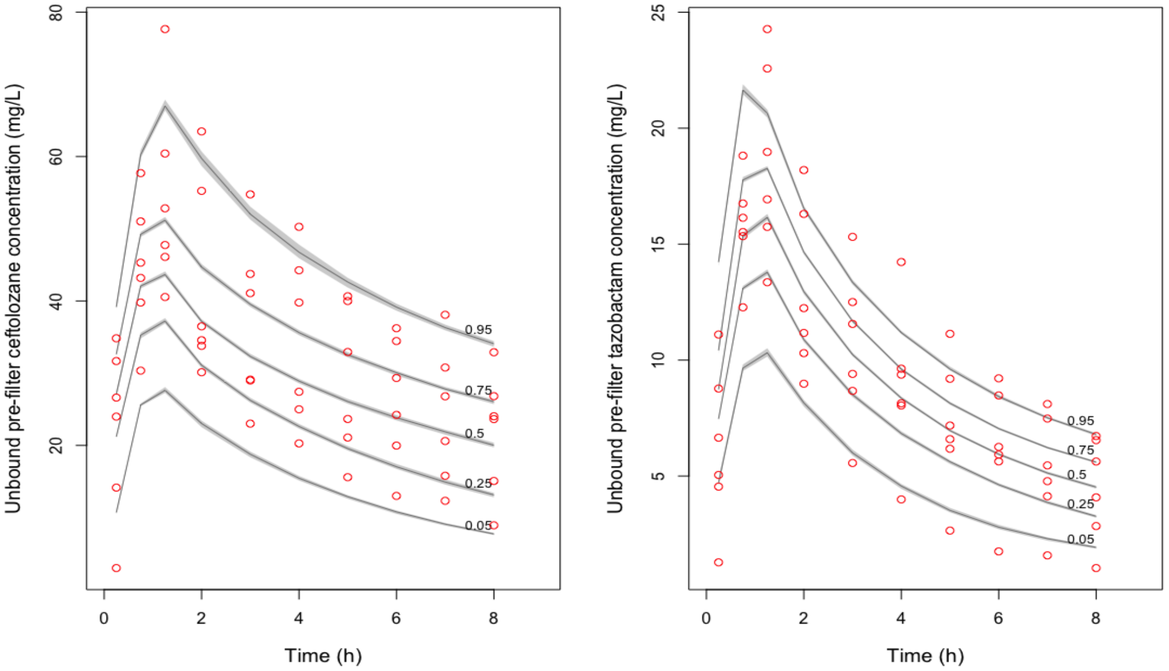

Supplement: Supplemental file 1 [file AAC.01655-19-s0001.pdf]
